# Mobile App for Assisting Patients With Type 1 Diabetes in Insulin Dose Adjustment: Development and Usability Study

**DOI:** 10.2196/99412

**Published:** 2026-11-06

**Authors:** Noor Kadhim Mohamed-Jawad, Ehab Mudher Mikhael, Haider Ayad Alidrisi

**Affiliations:** 1 Department of Clinical Pharmacy, College of Pharmacy, University of Basra, Basrah, Iraq; 2 Department of Clinical Pharmacy, College of Pharmacy, University of Baghdad, Bab Al-Muadham College Complex, Al-Waziriya District, Baghdad, 10071, Iraq, +964 7706216933; 3 Diabetes and Endocrinology, College of Medicine, University of Basra, Basra, Iraq; 4 Diabetes and Endocrinology, Faiha Specialized Diabetes, Endocrine and Metabolism Center (FDEMC), Basrah, Iraq

**Keywords:** type 1 diabetes, mobile app, insulin dose calculator, development and validation, Iraq

## Abstract

**Background:**

In resource-limited countries, most patients with type 1 diabetes (T1DM) use basal-bolus insulin therapy. Optimal dosing requires matching insulin to carbohydrate intake, premeal blood glucose levels, and physical activity—a complex process prone to error, particularly for patients with limited health literacy.

**Objective:**

This study aimed to develop and pilot-test a new mobile app designed to adjust bolus insulin doses based on the carbohydrate content of the meal, premeal blood glucose, and physical activity, tailored specifically for Iraqi patients with T1DM.

**Methods:**

To guide the app’s development, a literature search was conducted to identify key features in mobile apps for improving glycemic control in patients with T1DM. Evidence highlighted bolus insulin calculation, blood glucose tracking, and patient education as the most impactful features; consequently, these 3 functionalities were selected as the core components of the app. The scientific content of the app was based mainly on the latest American Diabetes Association guidelines and reviewed by 8 experts to ensure accuracy. After initial app prototype development, a cross-sectional pilot feasibility study was conducted at the Diabetes and Endocrine Center in Basrah, Iraq. A purposive sample of 10 health care professionals (5 endocrinologists, 2 dietitians, 2 clinical pharmacists, and 1 diabetes educator) and 10 patients with T1DM (aged ≥15 y) was recruited. The app was installed on participants’ smartphones, and standardized instructions on its functions and usage were provided. After a 10- to 14-day trial period, participants evaluated the usability, satisfaction, and perceived benefits of the app using newly developed and validated 3-point Likert scale questionnaires (one specific to patients and the other to health care professionals), alongside an optional open-ended question for qualitative feedback.

**Results:**

Positive feedback was reported across both groups. A favorable usability rating was expressed by 80% of experts and 90% of patients. Regarding app benefits, 90% of experts and nearly all patients considered it effective for adjusting insulin doses, improving treatment adherence, and reducing hypoglycemia. At least 70% of experts noted provider benefits (reduced workload and improved monitoring). All participants were satisfied and would recommend the app.

**Conclusions:**

The new app tested in this study demonstrated promising acceptability and usability among patients with T1DM and relevant health care professionals. However, given the small sample size, these findings should be interpreted as preliminary feasibility evidence. A larger, adequately powered clinical trial is warranted to confirm these results and evaluate the app’s clinical impact on glycemic outcomes.

## Introduction

Type 1 diabetes mellitus (T1DM) is a chronic disease characterized by autoimmune destruction of pancreatic β-cells, necessitating lifelong exogenous insulin administration [1]. Intensive insulin therapy, represented by multiple daily injections (MDIs) or continuous subcutaneous infusion through insulin pumps, is the standard care for achieving optimum glycemic control and reducing the risk of diabetes-related complications [2,3]. Effective insulin dosing, however, requires patients to match insulin dose to carbohydrate intake, premeal blood glucose (BG) levels, and anticipated physical activity [4]. This calculation is a complex process and is prone to error, especially for patients who lack health literacy and numerical skills [5]. While hybrid closed-loop insulin pumps can automate this process by linking continuous glucose monitoring systems with automated insulin delivery, access to such devices remains limited, especially in resource-limited countries [6,7]. Consequently, the majority of patients with T1DM in these regions continue to rely on manual dose calculation via MDI to maintain good glycemic control [8,9]. In Iraq and numerous other resource-limited countries, manual insulin dose calculation is a complex task compounded by limited health literacy [10-13], inadequate diabetes education [14], and unfamiliarity with carbohydrate counting methods [15]. As a result, a substantial proportion of patients in Iraq [16] and in other resource-limited countries [17,18] experience poor glycemic control, possibly due to administering suboptimal insulin doses [16].

Considering the significant potential of mobile apps to enhance health care delivery for patients with DM [19-21] and facilitate complex calculations [22], as well as the widespread availability of smartphones, even in resource-limited countries [13], the use of mobile health (mHealth) apps presents an attractive and accessible strategy to address challenges in insulin dose calculations for patients with T1DM in resource-limited countries. This in turn could improve glycemic management for such patients.

Many studies involved the development of apps facilitating insulin dose calculations. However, most remain institution-specific, are not free, or lack rigorous evidence-based validation [23-26]. More critically, most of these apps were not in Arabic and did not take into account the foods in Arabic cuisine [27-29], rendering them impractical for Iraqi patients. To address these identified gaps, this study had 2 main objectives: first, to design and develop a culturally tailored, Arabic mHealth app featuring a bolus insulin calculator, blood glucose tracking, and educational modules for Iraqi patients with T1DM; and second, to conduct a pilot feasibility study assessing the app’s usability, acceptability, and perceived clinical utility from the perspectives of both patients and health care providers.

## Methods

### Development of a Type 1 DM-Assisting Mobile App

According to the results of a recent systematic review evaluating the impact of mobile apps on patients with T1DM in resource-limited countries [29], these apps can offer greater benefits to patients with T1DM in such settings, particularly when they include multiple features such as bolus insulin dose calculation, a blood glucose tracking system, patient reminders, and educational support. Consequently, the current app was developed to incorporate all these features. Additionally, all information was written in Arabic, and the app was designed to include data on the most common foods in Iraqi cuisine to ensure its relevance and specificity for Iraqi patients with T1DM. The app development steps are summarized in Figure 1.

**Figure 1.**
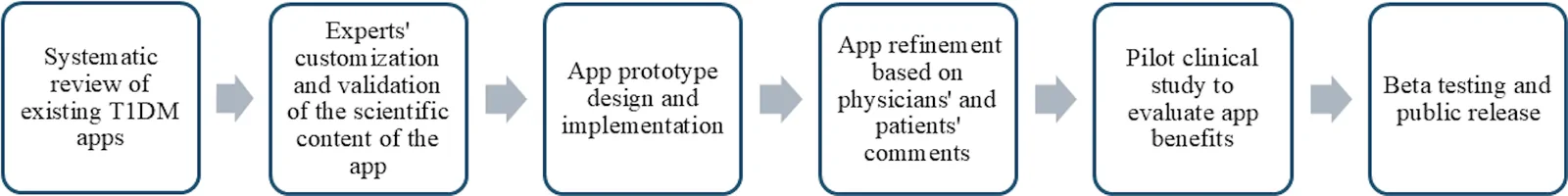
Sequential flowchart detailing the 6 core phases in the development and assessment of the type 1 diabetes (T1DM) medical app. The process begins with (1) systematic review of existing apps, (2) expert validation of scientific content, (3) prototype design and implementation, (4) iterative refinement based on physician and patient feedback, (5) pilot clinical study to evaluate benefits, and (6) beta testing leading to public release. This structured methodology ensured comprehensive validation and user-centered optimization prior to dissemination.

Scientific contents of the app were obtained from the latest American Diabetes Association guideline [30] and the latest version of an essential textbook in therapeutics, “Applied Therapeutics: The Clinical Use of Drugs” [31]. Moreover, names and contents of common foods in the Iraqi cuisine were obtained from “A Self-Management Guide for Type 2 Diabetes Mellitus Patients from Middle Eastern Countries” and “Iraqi Healthy Food Guide” [32,33]. The app was programmed by the Satyadri Innovations LLP company (India) using Flutter version 3.38 (Google LLC). The development process continued from Oct 2024 to Apr 2025.

### The Developed App Functionalities

The app was developed to include many functionalities. The user interface presents the main functions on a central dashboard on the app’s home screen, allowing for easy navigation [Figure 2]. The main app functions include (1) bolus insulin dose calculation and (2) a digital logbook for recording and tracking the trend in blood glucose measurements with graphical presentation of the BG trend. These records can be downloaded and shared with a health care provider; (3) educational information covering essential topics in T1DM self-management such as insulin injection techniques, counting, hypoglycemia management, insulin storage, and sick days management rules; (4) suggested meals with detailed carbohydrate content for common Iraqi breakfast, lunch, and dinner; and (5) a consultation contact number.

**Figure 2.**
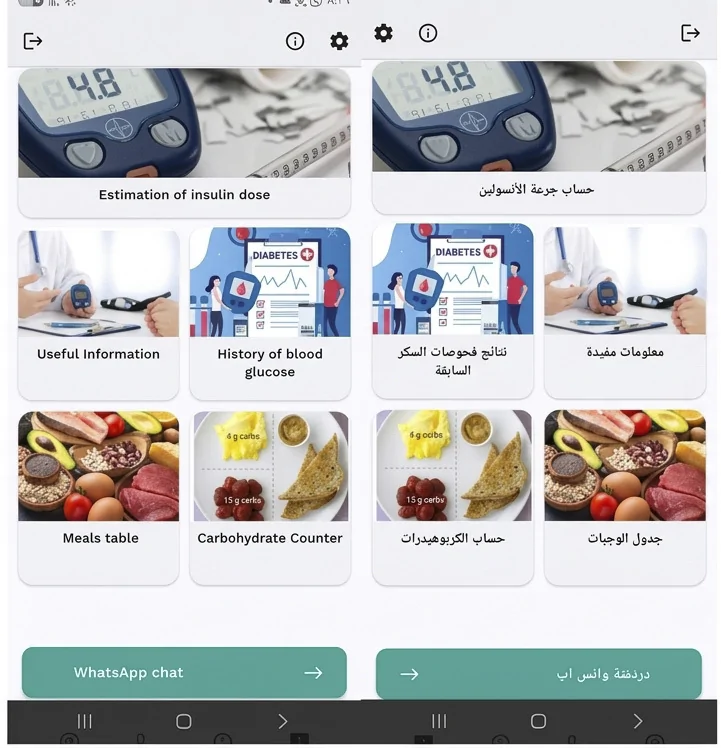
Bilingual mobile app homepages interface in English (left) and Arabic (right). The dashboards display localized navigation panels for key self-management features, including insulin dose estimation, blood glucose history, educational resources, a carbohydrate counter, dietary tables, and an integrated WhatsApp support chat.

The app also contains additional features such as reminder messages, which are sent 3 times daily (morning, afternoon, and evening) to prompt patients to use the app and to measure and record blood glucose level. Additionally, the app was designed to send alert messages to the user when alarming blood glucose levels are detected. These include instances of blood glucose exceeding the target threshold for 3 consecutive days and episodes of hypoglycemia. Upon detection, these notifications inform users of the situation and recommend an appropriate course of action. In addition, the digital logbook uses a color-coded system: blood glucose values above the target range are displayed in red with a “High” status note, while values within the normal range appear in green, facilitating immediate user awareness of glycemic excursions. The app was also designed to include a “Contact Us” button that allows patients to communicate directly with their physician in emergency situations, including sick days or frequent blood glucose fluctuations.

To protect sensitive patient information, the app implements robust privacy and security protocols. Patients must create a unique account with secure login credentials to access and manage their logbook. By default, patient-entered data is restricted from the health care provider view; clinical data sharing requires explicit consent, which users grant through an integrated opt-in privacy protocol.

### Bolus Insulin Dose Calculation

The app was designed to calculate the bolus insulin dose and then displays the recommended dose that the user should inject before a meal through a sequential process. To personalize dosing, the app was designed to enable users to select the type of insulin they are using from a list of commercially available insulins in Iraq and input their current prescribed doses. To ensure the accuracy of these calculations, users with above-target HbA_1c_ levels were required to perform intensive capillary blood glucose monitoring for 3 consecutive days and record their results in a logbook. The treating endocrinologist then reviewed these logbooks to adjust insulin doses as clinically indicated. If the total daily insulin dose was changed, an additional 1‐3 days of logbook checking was performed. These patients were then instructed to enter their newly adjusted doses into the app. Following dose entry by the patient, the app automatically calculates the total daily dose of insulin (TDDI). Using this individualized data (TDDI and insulin type), the app automatically derives the carbohydrate-to-insulin ratio (CIR) and correction factor (CF) using the validated clinical equations shown in Textbox 1 [31,32].

Textbox 1. **Mathematical equations used within the logical processing core of the medical app.**

A. Calculate the insulin dose based on carbohydrates in the meal.

(1) Totaldailydoseofinsulin(TDDI)=bolusinsulin+basalinsulin

(2) Carbohydrate-to-insulinratio(CIR)=X/TDDI

(X = 500 for those on rapid-acting insulin and 450 for those on regular insulin.)

(3) Insulindose=carbohydratemeal/CIR

B. Calculate the correctional insulin dose based on premeal blood glucose (BG).

(4) Correctionalinsulinfactor(CIF)=X/TDDI

where X = 1800 for patients using insulin analogues

and 1500 for those using regular insulin.

(5) Correctionalinsulindose=[CurrentBG−TargetBG]/CIF

C. Adjust insulin dose based on intended physical activity.

(6) Insulinreductionforexercise=calculatedinsulindose×%ofreduction

D. Calculate final insulin dose.

(7) Bolus insulin=meal insulin+correction insulin−insulin reduction for exercise

After that, the app calculates the insulin dose needed to cover carbohydrate in the meal by selecting the content from various types of foods, including popular Iraqi foods. The carbohydrate content of various food ingredients was estimated using multiple sources, including the US Department of Agriculture and the Quick Guide for Carbohydrate Counting provided by the National Diabetes Services Scheme [34,35]. The composition of each food item was analyzed by considering its ingredients and their respective proportions. The total carbohydrate content of each food item was then calculated based on its measured weight, determined using an electronic balance, in accordance with the typical and commonly used portion sizes in Iraq. All estimates were reviewed and validated by 2 registered Iraqi dietitians.

The app was designed to enable the user to select the type of food they intend to eat during a meal, starting from a list of general food categories, followed by selecting the specific food item within each category. The user can then specify the portion size of each selected food item they plan to consume. The app then automatically calculates the total carbohydrate content of the chosen food portion. The app then automatically calculates the needed insulin dose to cover carbohydrate in the meal by taking into account the type of insulin used by the user through specific equations (Textbox 1).

The app was designed to calculate correctional insulin dose by enabling the user to enter the premeal blood glucose level. It then automatically calculates the correctional insulin dose according to the premeal blood glucose level by taking into account the type of insulin used by the user through specific equations (Textbox 1B). To minimize user entry errors, the app only accepts blood glucose entries in the range between 70 and 500 mg/dl. Blood glucose entries below 70 mg/dl trigger an immediate hypoglycemia alarm advising corrective treatment: take 15 g of carbohydrate and recheck blood glucose after 15 minutes. The app uses the standard Iraqi unit mg/dL exclusively, eliminating conversion errors, and uses a numeric keypad interface to prevent invalid character inputs.

The effect of the intended physical activity on the bolus insulin dose is considered by selecting the type and duration of physical activity, if any, that is intended to be done postmeal. It then automatically calculates the needed percentage of the insulin dose (Table 1) that must be subtracted from the total calculated bolus insulin dose [36].

**Table 1.** Percentage reductions in insulin dosage categorized by exercise type and workload intensity.

Intensity of exercise (level of activity)	Examples^a^	Percentage of reduction in bolus insulin dose before exercise for exercise started within 90 min of consumption of the meal	
		Duration of exercise: 30 min	Duration of exercise: 60 min
Mild aerobic	Slow walking and grading	–25	–50
Moderate aerobic	Brisk walking, hiking, and cycling	–50	–75
Heavy aerobic exercise	Running, playing football, heavy agricultural work, shifting heavy loads, and construction labor	–75	—^b^

a The stratified adjustment values are adapted from established clinical guidelines [36]. Patients are instructed to match their planned physical activity—whether exercise or occupational—to the intensity level that best reflects their perceived exertion. For activities exceeding 60 minutes, additional adjustments may be required based on individual clinical assessment.

b Not available.

Finally, the needed bolus insulin dose is calculated through the following equation:

Bolus insulin dose = insulin dose needed to cover carbohydrate in the meal + correctional insulin dose –insulin dose reduced for the intended physical activity [37]. Details of all equations are shown in Textbox 1.

After presenting the recommended bolus insulin dose on the screen, the app was designed to provide additional guidance to users based on the selected insulin type by including (a) instructions on the appropriate timing for insulin injection and (b) a link to an educational video demonstrating proper insulin injection techniques.

### Validation of the Scientific Contents of the App

To ensure the validity of the scientific contents of the app—including the bolus insulin dose calculation equations, proposed app functions, and educational content—it was sent to a multidisciplinary panel of 8 experts (5 clinical pharmacists, 1 endocrinologist, and 2 dietitians). Experts were selected based on their clinical and academic expertise in diabetes care. All experts were instructed to evaluate the overall accuracy and comprehensiveness of the provided information using a 3-point Likert scale and to offer comments when applicable. A content validity ratio (CVR) was used, in which values ≥0.875 were considered acceptable [38]. All experts rated the accuracy and comprehensiveness as optimal, yielding a perfect CVR of 1.00 for all core scientific parameters. However, some feedback comments were received. All feedback was systematically compiled, reviewed, and incorporated into a revised app prototype through a consensus process involving the research team. Following this process, all experts unanimously agreed that the information was accurate, comprehensive, and clinically appropriate.

### Validation of the App Assessment Questionnaires

Two new questionnaires were developed by study authors based on ideas from previous literature [39,40]. The first questionnaire was developed in the English language and designed to assess the acceptability, quality, benefits, and ease of use of the app from the perspective of health care professionals using a 3-point Likert scale [39,40]. The second questionnaire was developed in the Arabic language and designed to assess the acceptability, benefits, and ease of use of the app from the perspectives of patients with T1DM using a 3-point Likert scale [39-42].

To check content validity of these questionnaires, they were sent to a panel of experts (3 endocrinologists and 4 clinical pharmacists), and they were asked to assess the relevance and clarity of the questionnaire items using a dichotomous response and comments when necessary. They were also asked to assess the comprehensiveness of the questionnaire as a whole. A CVR was used, in which values of 1 were considered acceptable [38]. All items in both questionnaires were considered relevant (CVR=1). Moreover, comments were received regarding the wording of several items to improve clarity, including 3 items in the first questionnaire and 2 items in the second questionnaire. An expert recommended the addition of 2 additional items to both questionnaires to improve comprehensiveness. Therefore, the questionnaires were revised according to the given comments.

The final expert questionnaire comprised 12 items across 4 domains (usability, benefit to patients, benefit to providers, and satisfaction), while the patient questionnaire comprised 10 items across 3 domains (usability, benefit to patients, and satisfaction). All items used a 3-point Likert scale: agree, neutral, and disagree. An optional open-ended question was included in both surveys for participant feedback. Full questionnaires are provided in Multimedia Appendices 1 and 2.

### Pilot Testing and Validation of the Developed App

#### Study Design

This study used a cross-sectional, pilot feasibility design and was conducted at the Endocrine Centre in Basra, Iraq, between May and June 2025. The pilot study followed current recommendations for feasibility and usability testing of mHealth interventions and served as an initial step to inform a subsequent larger-scale randomized controlled trial.

For pilot studies testing new apps, a sample size range of 10 to 20 participants is considered to be adequate to evaluate preliminary acceptability, usability, and technical issues [43]. Given the exploratory nature of this pilot study—aimed at assessing usability, acceptability, and perceived clinical utility of the developed app, along with generating qualitative feedback for iterative refinement—a sample of 10 patients and 10 experts was deemed appropriate and scientifically justified.

#### Participants

A purposive sample of health care professionals specializing in diabetes care was recruited from the Endocrine Center in Basra from May 1 to June 15, 2025. Eligible experts included 5 endocrinologists, 2 dietitians, 2 clinical pharmacists, and 1 diabetes educator nurse; all had at least 2 years of clinical experience managing patients with T1DM. Potential expert participants were identified from the Endocrine Center and were contacted directly to ask if they would participate.

Patients were consecutively recruited from the outpatient clinic of the Endocrine Center in Basra during the study period. Eligible patients were aged 15 years or older, with a confirmed diagnosis of T1DM for at least 1 year, on a stable insulin regimen for T1DM for at least 6 months prior to enrollment, and able to use a smartphone. Exclusion criteria included pregnancy or breastfeeding, hypoglycemia unawareness, and any psychological or physical disability. During routine clinical visits, the attending clinicians identified potentially eligible patients and informed them about the study. Individuals who initially expressed interest were given verbal explanations of the study’s objectives, methods, and voluntary nature of participation. The app was installed on each participant’s smartphone. All participants received instructions about its functions and usage.

#### Data Collection

The usability evaluation sessions were moderated by the first researcher, who was trained by the second researcher. The second researcher has extensive experience in conducting usability evaluations and has previously conducted usability studies with digital health interventions [44,45]. As this is the first mobile app developed for T1DM management in Iraq, none of the experts had prior formal experience in usability inspection methods. To ensure a standardized review process, the researcher provided a structured training and orientation session that included an overview of the study objectives, a demonstration of the app’s key functionalities and user interface, and a clear instruction on how to evaluate the content for accuracy, completeness, and relevance. Experts were given access to the app and asked to independently review the content over a 10- to 14-day period, providing feedback using a structured evaluation form (Multimedia Appendix 1). The paper-based form was distributed directly to the experts at the diabetes center, and the completed form was collected by the researcher at the end of the meeting.

For patients’ evaluation, participants were asked to use the app over a 10 to 14-day period and assess its features, clinical benefits, ease of use, navigation, time of use, and technical stability. Following this usage period, patients completed a self-administered paper-based structured questionnaire (Multimedia Appendix 2) during an interview at the diabetes center. Completed questionnaires were returned directly to the researcher at the end of the interview.

#### Data Analysis

Data were analyzed using Microsoft Excel. Continuous variables were presented as mean (SD). Categorical variables and participants’ responses (measured on a 3-point Likert scale) were presented as frequencies and percentages.

### Ethical Considerations

This study was conducted in accordance with the International Ethical Guidelines for Health-related Research Involving Humans from the Council for International Organizations of Medical Sciences (CIOMS) and the Belmont Report. Ethical approval was obtained from the Research Ethics Committee of the University of Baghdad–College of Pharmacy (approval number: REC0624485). The committee approved the use of verbal informed consent, as the study involved only an app usability assessment with minimal risk and did not include any experimental interventions or invasive procedures. All participants (health care providers and patients) provided verbal informed consent prior to enrollment after being fully informed about the study’s purpose and their right to withdraw at any time. To ensure privacy and confidentiality, all collected data were anonymized and restricted solely to the research team. Participants did not receive any financial compensation for their involvement in this study.

## Results

### Expert Validation of the Developed App

Ten experts validated the app prototype, with their demographic characteristics shown in Table 2. The expert evaluation of the T1DM app showed positive feedback across all domains, with high percentages of “Agree” ratings for most statements. In the usability domain, a large majority of the experts found the app easy to use and its details easy to understand, while a minority raised a concern about time efficiency. In the benefit to the patients domain, most experts agreed that patients with T1DM could benefit from the app and rated the app highly for optimizing insulin dose calculation, reducing hypoglycemia, and improving adherence to insulin, although a small subset felt it performed poorly in that aspect. Regarding benefit to health care provider, the app was strongly endorsed for improving communication; however, assessments of its ability to reduce physician workload were more mixed, with the majority agreeing, while some remained neutral and a few disagreed. In the satisfaction domain, all experts agreed they would recommend it to their patients. The numerical details of the expert ratings for the app are shown in Table 3. Finally, the recommendations from the expert panel included incorporating both standard and local portion measurements (dietitian), integrating graphical trends into the blood glucose log (endocrinologist), and simplifying educational content with visual aids (clinical pharmacist). All suggestions were incorporated into a revised prototype prior to pilot testing with patients with T1DM.

**Table 2.** Demographic characteristics of the experts (n=10) involved in the app validation.

Characteristic	Values
Age (y), mean (SD)	44.4 (7.84)
Sex, n (%)	
Male	7 (70)
Female	3 (30)
Duration of experience (y), mean (SD)	10.7 (7.38)
Specialty/academic degree, n (%)	
Endocrinologist	5 (50)
Dietitian	2 (20)
Clinical pharmacist	2 (20)
Educator nurse	1 (10)

**Table 3.** Expert consensus ratings (n=10) evaluating the clinical and functional utility of the mobile app.^a^

Domain and statement	Agree, n (%)	Neutral, n (%)	Disagree, n (%)
Usability			
The app is easy to use.	8 (80)	2 (20)	0 (0)
The details in the app are easy for patients with diabetes to understand.	8 (80)	2 (20)	0 (0)
Using the app does not take too much time for patients.	8 (80)	1 (10)	1 (10)
Benefit to the patients			
The app can optimize insulin dose calculation by patients with DM.	9 (90)	1 (10)	0 (0)
The app can enhance patients’ adherence to their insulin therapy.	9 (90)	0 (0)	1 (10)
The app may reduce the patients’ risk of developing hypoglycemia.	9 (90)	1 (10)	0 (0)
Patients with diabetes can benefit from this app in controlling their disease.	9 (90)	1 (10)	0 (0)
Benefit to health care provider			
The app reduces physicians’ workload in managing patients with diabetes.	7 (70)	2 (20)	1 (10)
The app improves communication between physicians and patients.	9 (90)	1 (10)	0 (0)
The app can help physicians in monitoring the blood glucose levels of their patients.	10 (100)	0 (0)	0 (0)
Satisfaction			
I recommend this app to my patients.	10 (100)	0 (0)	0 (0)

a Data are presented as frequency (n) and percentage (%). Evaluation scores are based on a 3-point Likert scale: agree, neutral, and disagree.

### Patients’ Evaluation: Usability, Utility, and Acceptance

Ten patients with T1DM completed the postintervention survey for the pilot study; their demographic characteristics are presented in Table 4. Patient evaluations reflected positive feedback across all assessment domains. In the usability/experience domain, the vast majority of patients found the app easy to use and time-efficient, and all agreed that the content was easily understood. In the benefits domain, all patients gave positive ratings for blood glucose tracking and enhanced adherence to insulin therapy, and all patients felt that the app improved their ability to manage blood glucose, while most believed it could reduce the risk of hypoglycemia. Finally, in the satisfaction domain, all patients expressed an intention to continue using the app and would recommend it to others (Table 5).

**Table 4.** Demographic characteristics of patient cohort (n=10) participating in the app evaluation phase.

Characteristic	Values
Age (y), mean (SD)	22.3 (6.97)
Sex, n (%)	
Male	6 (60)
Female	4 (40)
Duration of diabetes (y), mean (SD)	11.8 (7.46)
Educational level, n (%)	
Primary school	1 (10)
Secondary school	4 (40)
Diploma	1 (10)
College	4 (40)

**Table 5.** Patients’ ratings (n=10) of app usability and their acceptance of the app.^a^

Domain and statement	Agree, n (%)	Neutral, n (%)	Disagree, n (%)
Usability/experience			
The app is easy to use.	9 (90)	0 (0)	1 (10)
The details in the app are easy to understand.	10 (100)	0 (0)	0 (0)
Using the app does not take too much time.	9 (90)	0 (0)	1 (10)
Benefits			
The app helps me in BG^b^ tracking.	10 (100)	0 (0)	0 (0)
The app enhances my adherence to insulin therapy.	10 (100)	0 (0)	0 (0)
The app reduces the risk of developing hypoglycemia.	9 (90)	1 (10)	0 (0)
The app enhances my ability to control BG.	10 (100)	0 (0)	0 (0)
The app can improve my communication with specialists.	9 (90)	1 (10)	0 (0)
Satisfaction			
I intend to continue using the app.	10 (100)	0 (0)	0 (0)
I recommend this app to other patients.	10 (100)	0 (0)	0 (0)

a Data are presented as frequency (n) and percentage (%). Evaluation scores are based on a 3-point Likert scale: agree, neutral, and disagree.

b BG: blood glucose.

## Discussion

### Principal Finding

The current study highlights a logical progression from the development of a culturally adapted T1DM app to its pilot testing, integrating insights from both health care providers and patients with T1DM. Engaging both groups was critical to ensure clinical accuracy, usability, and practical relevance, consistent with established validation frameworks [46]. In this context, health care providers are best equipped to evaluate the app’s scientific content, clinical accuracy, and overall efficacy [47,48], whereas patients, as end users, are uniquely positioned to assess its usability, accessibility, and alignment with their daily self-management needs [48,49]. These findings confirm the app’s alignment with the clinical and practical needs of Iraqi patients with T1DM, and its ease of use establishes it as a tool for daily diabetes management.

### Comparison to Prior Work and Implications

The pilot study demonstrated strong agreement between experts and patients, especially regarding the app’s ease of use, which was attributed to its user-friendly design. Similar studies in Iraq have also found that users, regardless of educational background, perceive mobile apps as easy to use [44,45,50]. Most experts believed that the app’s details were clear for Iraqi patients with T1DM, a view supported by patients, who found the information understandable. This clarity was due to the use of Arabic, simplified data entry with food photographs, and avoidance of technical jargon. The study also found consensus that the app could quickly calculate insulin doses due to its automated design, which eliminates the need for manual calculations. This consensus on the app’s ease of use, clarity, and time efficiency confirms the app’s validity within the usability domain, thereby highlighting its suitability for use by Iraqi patients with T1DM.

Most experts agreed that the app effectively assists patients in administering accurate insulin doses—a process that many physicians are skeptical that patients can perform independently due to its complexity [51,52]. This confidence likely results from the app’s automated algorithm for insulin dose calculation, which considers key variables such as carbohydrate intake, postmeal exercise, and premeal blood glucose levels. Moreover, both experts and patients rated the app highly for reducing hypoglycemia risk. Unlike traditional methods that only consider carbohydrate intake and premeal glucose, this app also incorporates postmeal exercise—a variable that increases insulin sensitivity and hypoglycemic risk [53]. By integrating exercise into the algorithm, the app can better prevent or reduce hypoglycemia.

The high agreement among participants about the app’s role in enhancing patient adherence to insulin therapy is likely due to its reminder messages, which are known to boost medication adherence [54]. Moreover, the reduced risk of hypoglycemia and better glycemic control may serve as another motivator for enhancing patients’ adherence to insulin therapy [55-57].

Results also indicated that all patients found the app helpful for tracking blood glucose, while health care providers agreed that it facilitated monitoring of their patients’ blood glucose levels. Similar blood glucose monitoring features in other apps have been accepted by patients and providers [58-60], although none achieved universal acceptance. The strong support for this app may be due to its graphical representation of blood glucose trends, which helps users in identifying hyperglycemic or hypoglycemic patterns.

Another reported benefit of the developed app was its ability to improve communication between patients and health care providers, as confirmed by both groups. Although similar findings are limited in the literature, this benefit is logical and can be attributed to key app features: (1) educational information about diabetes and insulin therapy, which enhances patients’ knowledge and subsequently improves patient-provider communication [61]; (2) a blood glucose tracking system that can be easily reviewed by health care professionals; and (3) multiple communication channels, including email, phone, and WhatsApp, leading to more frequent and efficient interactions [62].

On the other hand, most of the participating health care providers considered the app to be effective in reducing the workload on physicians, possibly due to its ability to enhance patient self-management practices through its educational features and to optimize glycemic control through precise insulin dose calculations. Ultimately, these 2 features can reduce the workload on physicians and the burdens on the health care system by reducing the time needed to educate patients on self-management practices [63] and minimizing hospitalizations caused primarily by poor glycemic control [64].

The high satisfaction of patients and experts with the app, along with their willingness to continue using it and to recommend it to other patients with T1DM, may be attributed not only to its usability and clinical benefits but also to the fact that it is the first app specifically developed for Iraqi patients. This strong consensus supports the app’s overall validity.

### Beta Testing Outcomes and App Release

Following pilot testing, the app was prepared for public release through structured beta testing. This phase served as a critical bridge between clinical validation and public deployment, aligning with established mHealth research life cycles that position beta testing as a prerequisite for subsequent clinical trial evaluation [65]. Incorporating real-world user feedback and technical stress-testing prior to release ensures the app’s stability across diverse device configurations. The app complies with global clinical software standards, satisfying Apple App Review Guideline 1.4.1 and Google Play Health Policy requirements alongside international data protection protocols. The final version of the app was registered on Google Play and the Apple Store and became publicly available for download in August 2025. Annual update cycles will address emerging technical issues, user feature requests, and guideline updates, establishing a foundation for future randomized controlled trials to confirm its efficacy and safety. Future plans include submitting the app to the Iraqi Ministry of Health for clinical validation and adoption among Iraqi patients.

### Limitations

The findings of this pilot study are limited by the small sample size, which restricts the generalizability of the results. Additionally, the short post-intervention follow-up interview period may be insufficient for participants to comprehensively trial the app. All assessment parameters were subjective, relying solely on participants’ perceptions, without objective measures to confirm the benefits of the app. A further limitation is the manual entry of blood glucose values, which increases the risk of unintentional transcription errors. However, this design choice accounts for the high cost and limited availability of continuous glucose monitoring in Iraq, where conventional self-monitoring of blood glucose remains the standard of care. Future iterations of the app should prioritize Bluetooth connectivity with affordable glucometers to minimize transcription errors and enhance data reliability. Finally, the use of a 3-point Likert scale, while chosen to reduce cognitive load and maximize completion rates for the target population [66], inherently reduces response variance and statistical sensitivity, potentially constraining the ability to detect finer perceptual gradations relative to multipoint alternatives [67,68]. This compromise remains defensible within the present pragmatic context, though future investigations using broader Likert ranges may yield higher measurement resolution.

### Conclusions

The preliminary findings provide encouraging evidence that the new mobile app tested in this study is a feasible and acceptable decision-support tool for insulin dose adjustment among Iraqi patients with T1DM. Both end users and clinical experts highlighted the app’s high usability and supported its integration into routine care. However, these initial assessments focus on user perception rather than direct clinical impact. As a foundational feasibility study, this work establishes a clear rationale for a larger, adequately powered randomized controlled trial to evaluate the app’s true efficacy in optimizing glycemic control, lowering hypoglycemia risk, and improving patient quality of life.

## Supplementary material

10.2196/99412 Multimedia Appendix 1 App assessment questionnaire targeted at health care professionals.

10.2196/99412 Multimedia Appendix 2 App assessment questionnaire targeted at patients.

## Abbreviations

BG: blood glucose
CIF: correctional insulin factor
CIOMS: Council for International Organizations of Medical Sciences
CIR: carbohydrate to insulin ratio
CVR: content validity ratio
GCM: continuous glucose monitoring
MDI: multiple daily insulin
mHealth: mobile health
T1DM: type 1 diabetes
TDDI: total daily dose of insulin
