# Supplementary material for: Mobile App for Assisting Patients With Type 1 Diabetes in Insulin Dose Adjustment: Development and Usability Study
Source: JMIR Form Res. 2026 Nov 6;10:e99412. doi: 10.2196/99412 (PMC13743069; doi:10.2196/99412)
Supplement: Supplementary file 1 [file formative-v10-e99412-s001.docx]

**Appendix 1**

Application assessment questionnaire targeted to health care professional

| **Statement** | **Disagree** | **Neutral** | **Agree** |
| --- | --- | --- | --- |
| The application is easy to use |  |  |  |
| The details in the applications are easy for diabetic patient to understand. |  |  |  |
| Using the app does not take too much time for patients |  |  |  |
| The application can optimize insulin dose calculation by DM patients |  |  |  |
| The application can enhance patients' adherence to their insulin therapy |  |  |  |
| The application may reduce the patients' risk of developing hypoglycemia |  |  |  |
| Diabetic patients can benefit from this application in controlling their disease |  |  |  |
| The app reduces physicians’ workload in managing diabetes patients. |  |  |  |
| The application can help physicians in monitoring blood glucose level of their patients |  |  |  |
| The app improves communication between physicians and patients |  |  |  |
| I recommend this application to my patients |  |  |  |
| Give your comment, problem and/or suggestion to improve app. features? |  | | |
