# Supplementary material for: Mobile App for Assisting Patients With Type 1 Diabetes in Insulin Dose Adjustment: Development and Usability Study
Source: JMIR Form Res. 2026 Nov 6;10:e99412. doi: 10.2196/99412 (PMC13743069; doi:10.2196/99412)
Supplement: Supplementary file 2 [file formative-v10-e99412-s002.docx]

**Appendix 2**

Application assessment questionnaire targeted to the patients

| Statement | Disagree | Neutral | Agree |
| --- | --- | --- | --- |
| The application is easy to use |  |  |  |
| The details in the applications are easy for diabetic patient to understand. |  |  |  |
| Using the app does not take too much time for patients |  |  |  |
| The app helps me track my blood sugar levels regularly. |  |  |  |
| The app strengthens my adherence to my insulin treatment. |  |  |  |
| The app reduces my risk of hypoglycemia. |  |  |  |
| The app enhances my ability to control my blood sugar levels. |  |  |  |
| I believe the app will improve my communication with my doctor. |  |  |  |
| I intend to continue using this app. |  |  |  |
| I will recommend this app to people with diabetes. |  |  |  |
| Please share any comments, problems, or suggestions for improving the app's features, including any technical issues you encounter while using it. |  | | |
